# Simultaneous Correction of a Tibia Deformity and Non-union in Achondroplasia: A Case Report

**DOI:** 10.5704/MOJ.2207.018

**Published:** 2022-07

**Authors:** S Achudan, AXR Premchand, JS Low, J Decruz, SA Khan

**Affiliations:** 1 Department of Orthopaedics, Khoo Teck Puat Hospital, Singapore; 2 Department of Neurosurgery, Khoo Teck Puat Hospital, Singapore

**Keywords:** achondroplasia, tibia, non-union, deformity correction, hexapod

## Abstract

Tibial non-union with deformity in abnormal bone is rarely reported in literature. We report a case of a 65 years old male with a history of achondroplasia. The patient presented after a mechanical fall with an undisplaced right midshaft tibia fracture associated with pre-existing varus and procurvatum tibial deformities, which was initially managed non-operatively. However, after nine months he developed a painful non-union. Because of the symptomatic non-union as well as the pre-existing deformities, osteotomy of the tibia and fibula was performed with the application of a Truelok-Hexapod (TL-Hex, Orthofix) frame. We were able to achieve compression at the fracture site, and the software guided TL-Hex frame enabled gradual three-dimensional correction of the deformity. At six months, bony union and simultaneous correction of the tibia deformity were achieved. At two years, the patient was able to ambulate well without pain and perform his activities of daily living. We present a case of tibial non-union with pre-existing deformity in an achondroplasia patient successfully treated with a circular frame application.

## Introduction

Tibial shaft fractures are one of the most common long bone fractures in adults^1^. Tibia non-union is one of the known complications of shaft fractures that can cause significant patient morbidity and result in increased healthcare costs^2^.

Treatment options for tibia non-union include intramedullary nailing, dynamic compression plating, bone grafting, vascularised fibula grafting, electric stimulation, and external fixation with Ilizarov ring fixator. Although there are several methods of treating tibia non-union, studies have proposed the use of the Ilizarov ring fixator, especially in cases with existing tibial length deformities and angular defects.

While there has been literature on the treatment of tibia non-union with circular frames, to the best of our knowledge, the use of this fixation method has not been reported in patients with a background of achondroplasia.

We report a case of a 65 years old male with achondroplasia who had atrophic non-union of a mid-shaft tibia fracture with pre-existing tibia varus procurvatum deformity that was successfully treated with an application of the Truelok-Hex (TL-Hex) (Orthofix) circular fixator.

## Case Report

In early December 2015, a 65 years old male presented to the Khoo Teck Huat Hospital (KTPH) emergency department (ED) with right shin pain after a fall from standing height. Significantly, the patient had a history of achondroplasia. His other comorbidities included Type II Diabetes Mellitus, which was well controlled, as well as hypertension and hyperlipidaemia. The patient was a non-smoker and a non-drinker. With regards to his ambulatory function, before the fall, he was able to ambulate independently without aids and worked as a taxi driver.

On clinical examination, the patient had general features consistent with achondroplasia, namely frontal bossing, brachydactyly, and an exaggerated lumbar lordosis. He had a short stature of 1.3m in height as well as a marked genu varum deformity of the lower limbs. Local examination of the affected limb revealed a localised swelling over the right mid-shin with tenderness on palpation. Right knee and ankle range of motion (ROM) were full and pain-free. There was no vascular or neurological compromise of the right lower limb.

**Fig. 1:**
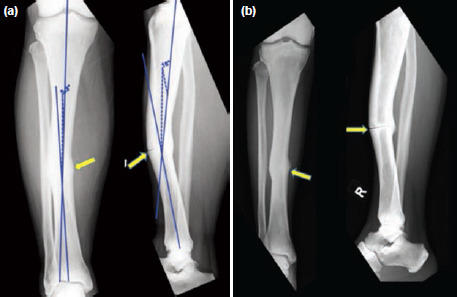
Pre-operative radiographs. (a) AP and lateral radiographs showing a fracture of the midshaft of the right tibia. The yellow arrow indicates the fracture site. The blue lines depict the angular deformities. On the AP radiograph, a varus angulation of 5.3° is seen. On the lateral radiograph, a procurvatum deformity of 16° is seen. (b) AP and lateral radiographs after nine months of follow-up showing non-union of the midshaft of tibia.

Anterior/posterior (AP) and lateral radiographs of the right tibia/fibula done in the ED showed an intact fibula with an incomplete midshaft fracture of the tibia as well as a procurvatum and mid shaft varus deformity (Fig. 1a). The patient was immobilised with an above-knee back slab and was discharged from the ED thereafter, with an outpatient appointment given to the Orthopaedic surgery clinic.

The patient was reviewed in the outpatient orthopaedic clinic two days later. Given the minimally displaced fracture pattern, with acceptable alignment, the patient was initially managed non-operatively. He was put on a cast and immobilised for about three months before starting on progressive weight-bearing of the right lower limb. However, after nine months of follow-up with conservative management, the patient still had symptoms of persistent pain in the right mid-shin region. Repeat radiographs showed non-union of the right tibia fracture (Fig. 1b).

As the patient had developed painful non-union, he was counselled for operative management to treat this complication. In view of the pre-existing procurvatum and varus deformity coupled with a non-union of the tibia, an osteotomy of the fibula and external fixation of the tibia with a ring fixator was considered the best fixation option. The ring fixator would allow for management of the non-union and for correction of the deformity. Intra-medullary nailing and compression plating were other treatment options that were considered. However, as part of our surgical goals entailed correcting the multi-planar deformity, external fixation with a circular frame was deemed to be the best option. On our pre-operative planning, we also noted that the patient had a narrow tibia canal. Moreover, with technical considerations such as the different anatomy of the patient, as well as a history of diabetes, the option of a circular frame provided less soft tissue and periosteal damage to improve overall healing at the non-union site.

The patient subsequently underwent osteotomy of right tibia and fibula and an application of the TL-Hex (Orthofix) frame on the 16th of October 2016. With the frame, we were able to achieve stable compression of the fracture site. Through software guidance and adjustment of the telescopic struts, the frame also enabled gradual three-dimensional correction of his deformity.

The operation was done under general anaesthesia with the patient positioned in supine. An oblique fibula osteotomy was performed with an oscillating saw through a lateral incision at the same level of the tibia shaft fracture.

**Fig. 2:**
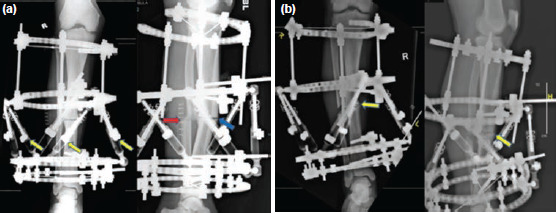
Post-operative radiographs post frame insertion. (a) Immediate post-operation AP and lateral radiographs. Yellow arrows on the AP radiograph indicate the adjustable telescopic struts. The red arrow on the lateral radiograph indicates the oblique osteotomy that was made to the fibula. The blue arrow on the lateral radiograph shows the compression at the fracture site. (b) AP and lateral radiographs at three months post-operation with the hexapod frame still on. The yellow arrows show the bridging callus formation at the fracture sites.

Two 160mm hexapod rings were assembled and positioned across the non-union site of the tibia. The hexapod rings were then fixed to the tibia with two half pins proximally and two half pins distally. The distal segment was reinforced with an additional 160mm full ring while the proximal segment was reinforced with an additional 160mm 5/8 ring and fixed using crossed wires (Fig. 2a). An olive wire inserted across the proximal tibia/fibula joint to prevent any subluxation during correction. Lastly, the tibia non-union site was freshened up with multiple drill holes across this area, to stimulate an inflammatory response. No bone graft was added.

Post-operatively, the patient was allowed weight bearing as tolerated with the frame. The pin and wire sites were dressed daily with chlorhexidine-soaked gauze per our hospital protocol. The patient was also educated on the daily adjustment of struts which would assist in the gradual correction of the bony deformity. This hexapod programme produced a treatment regimen for the patient to follow which allowed for gradual deformity correction to occur at 1mm per day. This correction was started after a latent period of 10 days. After the patient was competent in adjusting the struts himself and performing daily dressing to the pin sites, he was discharged from the hospital with regular reviews in the outpatient clinic to monitor for pin site infections and adjustment of the struts. Radiographs at three months post-operation showed good healing at the fracture site as well as gradual correction of the tibia mid shaft varus and procurvatum deformities. At three months post-operation, there were signs of clinical union, with pain-free ambulation and minimal tenderness at the fracture site (Fig. 2b). The patient was then scheduled for removal of the external fixator after clinical and radiological union was confirmed.

**Fig. 3:**
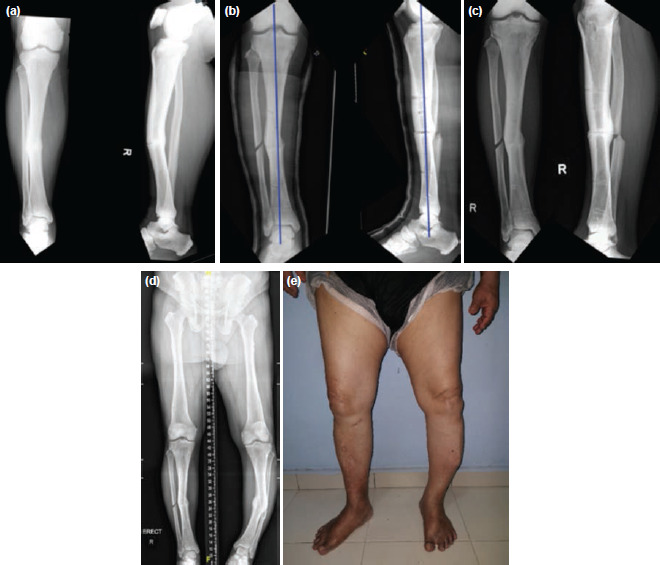
Post-operative radiographs. (a) and (b) Comparison pre-operative (a) and post-operative (b) radiographs. The post-operative radiographs in (b) show complete bony union and correction of the procurvatum and varus deformities are demonstrated by the blue lines indicating the correction of the mechanical axis. (c) AP and lateral radiographs two years after index operation, showing solid union at the non-union site as well as correction of the varus and procurvatum deformities. (d) Long leg film showing the deformity correction at the right lower limb. (e) Clinical photo of the patient’s lower limb with the operated right lower limb demonstrating a comparatively normal mechanical alignment.

The patient was put in a below-knee walking cast for 6 weeks after the removal of the circular ring fixator and allowed to fully weight bear. The decision was made to remove the cast after post-operative radiographs showed no displacement of the deformity or mal reduction at the fracture site (Fig. 3a and 3b). At two years follow-up, he was able to ambulate pain-free without aid and was able to cope with daily activities, including his premorbid occupational duties. The radiographs showed complete bony union and correction of varus and procurvatum deformities (Fig. 3c). A long leg film was done at four years of follow-up and revealed adequate deformity correction of the right lower limb (Fig. 3d) and revealed that he had a more neutral alignment to his right lower limb clinically (Fig. 3e).

## Discussion

Though there has not been any established consensus on the optimal treatment for tibia non-union, the use of circular external fixators in non-union management has gained popularity in recent years. In addition to providing excellent stability against translational and rotational forces, circular external fixators also create a low strain environment for secondary bone fracture healing to take place across the non-union site. Furthermore, several studies have also demonstrated the use of circular frames for successful deformity correction of the tibia as well^3^. Thus, the application of circular frames for both non-union and deformity correction has been well documented.

We have demonstrated the use of a hexapod circular frame in the management of a tibia non-union with a known varus and procurvatum deformities.

In our case report, a unique aspect was that this surgical technique was applied to a patient with achondroplasia. A technical challenge in our case was that our patient was short and thus accurate pre-operative templating was done to ensure that correct sizes of the rings and struts were obtained. A literature review was performed on PubMed, Elsevier, Medscape and Google Scholar electronic. Keywords such as ‘achondroplasia’, ‘tibia’, ‘non-union’, ‘deformity’ were used in our search. In our review, we found only three other case studies on fracture fixation in adult patients with achondroplasia, all of which highlighted the technical complexities of performing surgery in this patient profile as well as the paucity of data on it^4^.

Common orthopaedic surgeries done for achondroplasia patients include limb lengthening procedures and deformity correction. Arthroplasty procedures such as hip replacement operations have also been well documented in this patient profile as well. Though these various operations have been well documented, there is little data on fracture fixation and non-union management specific to patients with achondroplasia. Fracture occurrence and treatment measures are mostly discussed as part of post-operative complications after limb lengthening procedures.

With regards to the patient’s non-union, we believe that there were several factors that contributed to it. Firstly, the initial fracture pattern was a transverse tibia shaft fracture with an intact fibula. The intact fibula prevented the tibia from uniting. An intact fibula tends to distract a high percentage of load and resist compression at the tibia fracture site, reducing effective compression needed for fracture healing. Secondly, in our case, the patient’s tibia was deformed pre-injury due to achondroplasia. We thus postulate that the pre-existing genu varum and procurvatum deformities in our patient may have led to additional mechanical strain on the fracture site leading to non-union. In the non-deformed tibia, the area of tension is usually at the anterior surface, while the posterior-medial surfaces are in compression. In a tibia that has genu varum and procurvatum, we hypothesise that these forces are magnified. Forces beyond physiological loading may lead to strain at the fracture site and contributed to the tibia non-union.

Murphy *et al*^5^, described the treatment of a distal femur fracture in an elderly female patient with achondroplasia. He mentioned the need for meticulous templating of available fixation options to cater to the anatomical challenges posed by having a patient with achondroplasia such as poor bone quality and bone stock. This similar meticulous planning process was also required in our case given that we had to address issues of non-union and deformity correction whilst also dealing with concerns of poor bone quality as well.

There is a paucity of data on the technical challenges faced when dealing with fracture management for patients with achondroplasia. The optimal surgical intervention for tibial shaft non-union remains controversial. Optimal treatment should consider the risks and factors causing non-union and address them accordingly. In this case report, as the patient had both stability and deformity issues, the decision was made for TL-Hex (Orthofix) fixator to achieve compression at the fracture site and gradual correction of tibial shaft deformity. This device provides rigid fixation that allows for weight-bearing immediately after surgery. It is also a relative stability construct thus allowing micromotion at the fracture site to stimulate callus formation.

The circular hexapod fixator can be utilised as a means of definitive treatment for tibial non-union. It is a safe and reliable method for achieving bony union as well as deformity correction. We recommend circular frames for patients with achondroplasia due to its versality in dealing with both non-union and deformity correction.
